# Supporting data for identification of biosurfactant-producing bacteria isolated from agro-food industrial effluent

**DOI:** 10.1016/j.dib.2016.03.058

**Published:** 2016-03-19

**Authors:** Mohamad Ali Fulazzaky, Shakila Abdullah, Mohd Razman Salim

**Affiliations:** a Centre for Environmental Sustainability and Water Security, Research Institute for Sustainable Environment, Universiti Teknologi Malaysia, UTM Skudai, 81310 Johor Bahru, Malaysia; b Faculty of Civil Engineering, Universiti Teknologi Malaysia, UTM Skudai, 81310 Johor Bahru, Malaysia; c Faculty of Science, Technology and Human Development, Universiti Tun Hussein Onn Malaysia, Parit Raja, 86400 Batu Pahat, Johor, Malaysia

## Abstract

The goal of this study was to identify the biosurfactant-producing bacteria isolated from agro-food industrial effluet. The identification of the potential bacterial strain using a polymerase chain reaction of the 16S rRNA gene analysis was closely related to *Serratia marcescens* with its recorded strain of SA30 “Fundamentals of mass transfer and kinetics for biosorption of oil and grease from agro-food industrial effluent by *Serratia marcescens* SA30” (Fulazzaky et al., 2015) [1]; however, many biochemical tests have not been published yet. The biochemical tests of biosurfactant production, haemolytic assay and cell surface hydrophobicity were performed to investigate the beneficial strain of biosurfactant-producing bacteria. Here we do share data collected from the biochemical tests to get a better understanding of the use of *Serratia marcescens* SA30 to degrade oil, which contributes the technical features of strengthening the biological treatment of oil-contaminated wastewater in tropical environments.

**Specifications Table**

**Table:** 

Subject area	Water
More specific subject area	Water Chemistry and Microbiology
Type of data	Tables and figures
How data was acquired	A polymerase chain reaction of the 16 S rRNA gene analysis. The biochemical tests i.e., biosurfactant production, haemolytic assay and cell surface hydrophobicity.
Data format	Filtered and analysed
Experimental factors	The adapted *Serratia marcescens* SA30 and non-adapted *S. marcescens* SA30 were tested for their ability to degrade oil.
Experimental features	The agro-food industrial effluent (AFIE) samples were collected from an agro-food factory and then the biosurfactant-producing bacterial strains were isolated from the AFIE outlet of industrial process inside the agro-food factory, using the benificial strain for further tests.
Data source location	Skudai, Johor Bahru, Malaysia
Data accessibility	Data are within this article.

**Value of data**

**Table:** 

• This data information provides isolation and identification of the beneficial strain of biosurfactant-producing bacteria to degrade oil.
• Data can be used for comparative studies related to the removal oil and grease from wastewaters in the tropical environments.
• Further analysis of the data should allow a new insight into the ability of *Serratia marcescens* SA30 to degrade oil and grease that contributes the technical features of strengthening the biological wastewater treatment.

## 1 Data

Two different AFIE samples collected from an agro-food factory in Johor state of Malaysia containing high concentration of O&G were presented in Supplementary Table 1. From ten isolated bacterial colonies, the only one bacterial strain of AF01-O that having a MATH percentage of greater 80% (Supplementary Fig 1) to be feasibly selected it for reusing with further experiments to generate the other related data [2]. The biosurfactant production does not appear in Erlenmeyer flask without added *Serratia marcescens* SA30 (Supplementary Fig 2a) and clearly appears from experiment with added *Serratia marcescens* SA30 (Supplementary Fig 2b) observed after 72 h [3], [4], [5], the zone haemolysis produced by *Serratia marcescens* SA30 was classified as a “+” weak haemolysis (Supplementary Fig 2c) [6], and the percentage of MATH almost two times higher for adapted (81%) than non-adapted (42%) *Serratia marcescens* SA30 was presented (Supplementary Fig 2d) [7].

## 2 Experimental design, materials and methods

### 2.1 Culture media

Four culture media were used for data collection, i.e. (1) nutrient broth (Merck, Germany), (2) minimal medium, (3) nutrient agar (Merck, Germany) and (4) blood agar (Oxoid Ltd., Hampshire, UK). For the nutrient broth, 8-g nutrient broth was diluted in 1 L of distilled water. The minimal medium consisted of 3 g (NH_4_)_2_SO_4_, 0.5 g KH_2_PO_4_, 0.1 g KCl and 0.5 g MgSO_4_.7H_2_O per L of distilled water. For the nutrient agar, 20-g nutrient agar was diluted in 1 L of distilled water. The blood agar consisted of 40-g blood agar and 7% (v/v) sterile cow blood per L of distilled water with a neutral pH of 7. Each culture medium was placed in 1-L Schott bottle and then sterilised by autoclaving at 121 °C and 103 kPa for 20 min.

### 2.2 Agro-food industrial effluent

The AFIE sample used to produce the data was collected from an agro-food factory located at Rengit, 90 km from Johor Bahru, the state capital of Johor, Malaysia. Sampling point selected for isolating the strains of biosurfactant-producing bacteria was at an outlet of the industrial processes inside the agro-food factory. For the isolation purpose, 1 L of the effluent sample was filled in a 1-L sterilised Schott bottle. The sample being transported to testing laboratory must be maintained at an appropriate temperature and kept in an ice-packed container.

### 2.3 Isolation and screening of the bacterial strains

The first step for collecting data was to perform the isolation of the bacterial strains. A 2.5-mL AFIE sample was transferred from the Schott bottle to a 22.5 mL of nutrient broth in a 250-mL Erlenmeyer flask as active culture and made to order with three active cultures. Each live active culture was then shaken at 25, 30 and 37 °C with constant rotation (200 rpm). After the incubation periods of 24 and 48 h, one loop of aech incubated active culture was inoculated by streaked plate technique onto a sterilised nutrient agar plate and then incubated again at 25, 30 and 37 °C for selecting the isolation of a single colony. A few handred bacterial colonies isolated from the AFIE sample were examined based on the same morphological features i.e. form, margin, surface, eleviation, colour, opacity and gram staining to having ten single colonies, according to the Bergey׳s Manual of Determinative Bacteriology [8]. The typical colony was carefully selected streaked again onto a sterilised nutrient agar plate and incubated at 25, 30 and 37 °C for obtaining a pure culture. In general, the cultures grown effectively at 30 °C should never be kept for more than 15 d. Ten single colonies originally coming from AFIE were then successfully isolated from the nutrient agar, and the live active cultures were kept in a Luria Bertani (LB)-glycerol medium for the further uses.

The following steps were used for screening the biosurfactant-producing bacterial strains for collecting valid data, such that: (1) one loop of each isolated strain was transferred from the LB-glycerol medium to a 250-mL Erlenmeyer flask containing 25 mL of sterile nutrient broth and then shaken at 200 rpm for 24 h, (2) 2.5 mL of the overnight culture was added into 22.5 mL of nutrient broth in a 250-mL Erlenmeyer flask and then shaken at 200 rpm for 24 h, (3) to eliminate the contaminants of glycerol, it needs to repeat the procedure of step-2 at least three times, (4) 2.5 mL of the active culture was transferred from the free glycerol nutrient broth to a 250-mL Erlenmeyer flask containing 22.5 mL of sterile minimal medium and 1% (v/v) of palm oil and then cultivated at 30 °C for 24 h with rotation at 200 rpm, and (5) 3 mL of the bacterial strains was transferred from the minimal medium to a cuvette for the observation of cell growth identified by optimal optical density (OD_600_), measured by the DR5000 Spectrophotometer at a wave length of 600 nm. Even though ten bacterial strains, noted “agro-food oil-contaminated bacterial strain” and then abbreviated as “AF(test number)-O” i.e. AF01-O, AF02-O, AF03-O, AF04-O, AF05-O, AF06-O, AF07-O, AF08-O, AF09-O and AF10-O, were tested to screen their participants for the selection of high potential biosurfactant-producing bacteria, the only one strain of AF01-O has shown an optimal OD_600_ of greater 1.0 to be feasibly selected it as “non-adapted bacterial strain” for potential use in the further experiments to produce the other related data.

### 2.4 Identification of the genotype of bacterial strain

For obtaining the data of bacterial strain genotype, the extraction of DNA using the GF_1 DNA Extraction Kit was carried out at the Vivantis Technologies Sdn Bhd Laboratory of the Revongen Corporation Centre, Selangor, Malaysia. A polymerase chain reaction (PCR)-mediated amplification of the 16S rDNA genes and sequence determination of the bacterial strain were performed by the Vivantis Technologies Sdn Bhd Laboratory to follow the manufacturer׳s instructions. The genotype of AF01-O strain was identified as “*Serratia marcescens*” by using a polymerase chain reaction-based analysis of 16S rRNA genes. The submitted sequence appears in the GenBank nucleotide sequences databases under accession number KF686740 [1] for such a bacterial strain of *Serratia marcescens* with recorded strain SA30 and thus named as “*Serratia marcescens* SA30”, full sequence of 1398 nucleotides, and accession date of 4 December, 2013.

### 2.5 Adaptation of Serratia marcescens SA30 strain

The adaptation of *Serratia marcescens* SA30 to AFIE was performed to compare the data related to the adapted and non-adapted bacterial strains. One loop of the *Serratia marcescens* SA30 strain was transferred from the LB-glycerol medium to a 250-mL Erlenmeyer flask containing 25 mL of sterile nutrient broth. The active live culture was adjusted to a gradual change in its environment by increasing the concentration of AFIE from 0 to 20 to 40 to 60 to 80 and to 100% (v/v) during a period of 15 d. Then the isolation of bacterial strain, called it as “adapted *Serratia marcescens* SA30”, was carried out with the same procedure used for that of non-adapted *Serratia marcescens* SA30 and kept in a LB-glycerol medium for the further tests to produce the other related data.

### 2.6 Measurement of biosurfactant production

The resulting isolates contained exclusively bacterial strains, making them acceptable for microbial hydrocarbon degradation use, and produced a larger amount of biosurfactants in a minimal medium composed of palm as the sole carbon source. The adapted bacterial strain was cultured at 30 °C in a minimal medium with palm oil concentration of 5% (v/v) and pH 7. The experiments were carried out in duplicate in a 250-mL Erlenmeyer flask containing 50 mL of basal mineral salt medium. The flasks were placed on an orbital shaker (Certomat, B. Braun) at 200 rpm and 30 °C for 72 h. The culture broth was centrifuged under 4 °C at 10,000 rpm for 10 min and extracted by blending in chloroform-methanol (2:1, v/v). The solvents were removed by rotary evaporation and the resultant residue obtained was defined as “crude biosurfactant”. Weight of crude biosurfactant was expressed in terms of mg mL^−1^ (dry weight) to having the data of biosurfactant production.

### 2.7 Haemolytic assay

The isolate of adapted bacterial strain was streaked on a blood agar plate and then incubated at 37 °C for 72 h. The blood agar plate became cloudy as a result of the particulate biosurfactant present in the culture medium, enabling visual inspection of clearing zone around biosurfactant-producing bacterial colonies. The diameter of clear zone depends on the concentration of biosurfactant produced by the bacterial colonies [6], [9]. Haemolytic activity might be expressed in term of the ratio of colony diameter to outer diameter of clearing zone. Colony and halo diameter were measured with a ruler that the data classification of haemolytic zone diameter includes the following: (1) “−” very weak haemolysis that haemolytic activity showed no clear halo formation in response to the presence of pam oil in culture medium, (2) “+” weak haemolysis that haemolytic activity had the haemolysis diameter less than 1 cm, (3) “++” moderate haemolysis that haemolytic activity had the haemolysis diameter between 1 and 2 cm, (4) “+++” strong haemolysis that haemolytic activity had the haemolysis diameter between 2 and 3 cm, and (5) “++++” very strong haemolysis that haemolytic activity had the haemolysis diameter greater than 3 cm [6].

### 2.8 Measurement of cell surface hydrophobicity

The measurement of cell surface hydrophobicity (CSH) using a MATH indicator might distinguish microbial subpopulations with different ability to adhere to the hydrocarbon phase [7], [10]. The adapted and non-adapted bacterial cells were harvested from minimal medium by centrifugation at 9,500 rpm for 5 min and resuspended in 0.01-M potassium phosphate buffer (pH 7) to get to OD_600_=0.5. Then 0.5-mL palm oil was added to 5 mL of cell suspension and mixed using a vortex mixer (Thermolyne Maxi Mixer II) for 30 s. After the suspensions settled at room temperature for 15 min, the absorbance of aqueous phase was measured by the DR5000 Spectrophotometer at a wave length of 600 nm. The percentage of microbial adhesion to hydrocarbon was calculated to having the data of MATH percentage, as follows:

(1) $$\%\text{MATH}=1\;-\;\frac{{\mathrm{OD}}_{600}\text{of}\;\text{aqueous}\;\text{phase}\;\text{of}\;\text{cell}\;\text{suspension}\;\text{after}\;\text{mixing}\;\text{with}\;\text{palm}\;\text{oil}}{{\mathrm{OD}}_{600}\text{of}\;\text{initial}\;\text{aqueous}\;\text{phase}\;\text{of}\;\text{cell}\;\text{suspension}}\times 100\%$$
