# Supplementary material for: Supporting data for identification of biosurfactant-producing bacteria isolated from agro-food industrial effluent
Source: Data Brief. 2016 Mar 19;7:834–8. doi: 10.1016/j.dib.2016.03.058 (PMC4816861; doi:10.1016/j.dib.2016.03.058)
Supplement: Supplementary file 1 — Supplementary material [file mmc1.docx]

**Conflict of Interest:** The authors declare that they have no conflict of interest.
