# Supplementary material for: Supporting data for identification of biosurfactant-producing bacteria isolated from agro-food industrial effluent
Source: Data Brief. 2016 Mar 19;7:834–8. doi: 10.1016/j.dib.2016.03.058 (PMC4816861; doi:10.1016/j.dib.2016.03.058)
Supplement: Supplementary file 2 — Supplementary material [file mmc2.zip › 2016 data in brief Figure 1 .docx]

**Figure 1** Percentage of MATH for ten tested bacterial strains
