# Supplementary material for: Supporting data for identification of biosurfactant-producing bacteria isolated from agro-food industrial effluent
Source: Data Brief. 2016 Mar 19;7:834–8. doi: 10.1016/j.dib.2016.03.058 (PMC4816861; doi:10.1016/j.dib.2016.03.058)
Supplement: Supplementary file 2 — Supplementary material [file mmc2.zip › 2016 data in brief Figure 2 .docx]

| 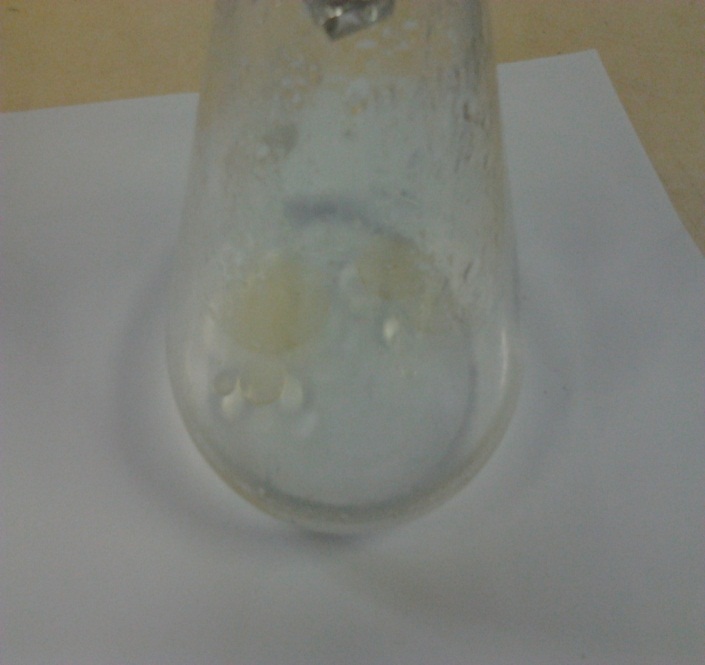  (a) | 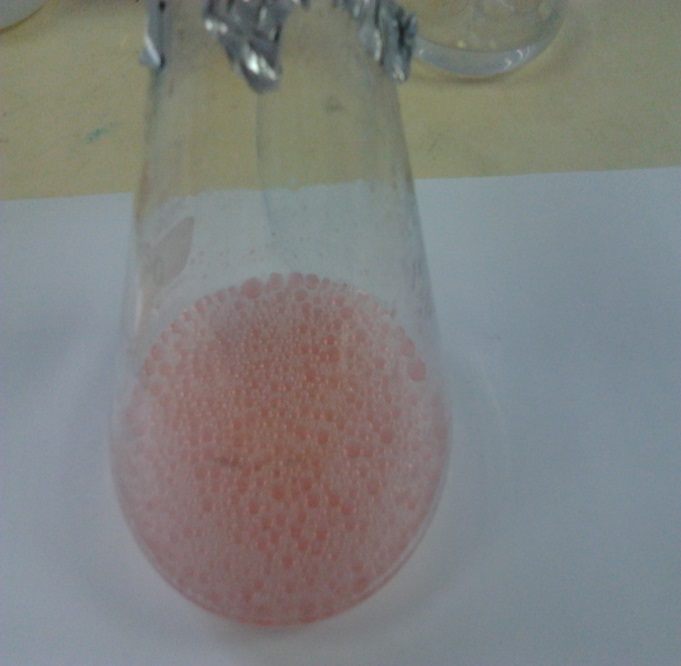  (b) |
| --- | --- |
| 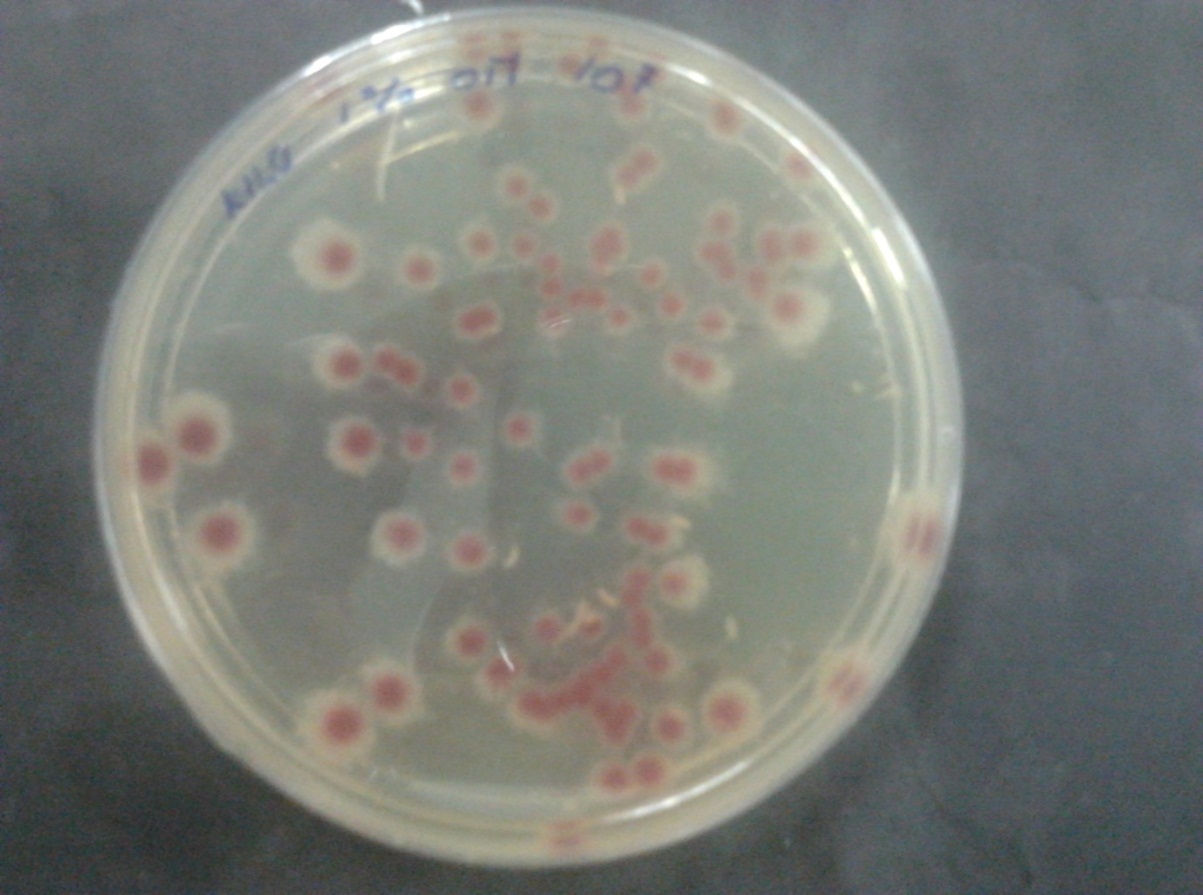  (c) |  |

**Figure 2** Identification of the biosurfactants; **(a)** the control test of biosurfactants production without added *Serratia marcescens* SA30, observed after 72 h, **(b)** the sample test of biosurfactants production with added *Serratia marcescens* SA30, observed after 72 h, **(c)** the haemolytic zone (clear zone) produced by *Serratia marcescens* SA30, and **(d)** the percentage of MATH with **(i)** adapted *Serratia marcescens* SA30 and **(ii)** non-adapted *Serratia marcescens* SA30.
