# Supplementary material for: Supporting data for identification of biosurfactant-producing bacteria isolated from agro-food industrial effluent
Source: Data Brief. 2016 Mar 19;7:834–8. doi: 10.1016/j.dib.2016.03.058 (PMC4816861; doi:10.1016/j.dib.2016.03.058)
Supplement: Supplementary file 2 — Supplementary material [file mmc2.zip › 2016 data in brief Table 1 .docx]

**Table 1** Main characteristics of the agro-food industrial effluent

| No | Parameter | unit | EO^(a)^ | DS^(b)^ |
| --- | --- | --- | --- | --- |
| 1 | Temperature | ^o^C | 28.9 | 28.9 |
| 2 | pH | - | 5.46 | 5.73 |
| 3 | DO | mg L^-1^ | 3.5 | 4.5 |
| 4 | BOD | mg L^-1^ | 2800 | 2875 |
| 5 | COD | mg L^-1^ | 5625 | 4125 |
| 6 | TOC | mg L^-1^ | 9485 | 7412 |
| 7 | NH_4_^+^ | mg L^-1^ | 146.1 | 181.6 |
| 8 | SS | mg L^-1^ | 7800 | 9100 |
| 9 | TDS | mg L^-1^ | 393 | 390 |
| 10 | O&G | mg L^-1^ | 42974 | 19395 |
| Remarks that: ^(a)^EO is the effluent outlet of the industrial process inside the agro-food factory and ^(b)^DS is the drainage system located outside the agro-food factory before discharging into the river. | | | | |
